# Predictive Value of Apelin-36 for No-Reflow Phenomenon in STEMI Patients

**DOI:** 10.3390/life16010094

**Published:** 2026-01-08

**Authors:** Xhevdet Krasniqi, Xhevat Jakupi, Josip Vincelj, Gresa Gojani, Petrit Çuni, Labinot Shahini, Adriana Berisha, Kreshnik Jashari, Blerim Berisha, Aurora Bakalli

**Affiliations:** 1 Department of Internal Medicine, Medical Faculty, University of Prishtina “Hasan Prishtina”, 10000 Prishtina, Kosovogojanigresa@gmail.com (G.G.); petritcunii89@gmail.com (P.Ç.);; 2 Department of Cardiology, University Clinical Center of Kosova, 10000 Prishtina, Kosovo; 3 Department of Cardiovascular Medicine, Dubrava University Hospital, HR-10000 Zagreb, Croatia; 4 Kreisklinik Roth, Kardiologie und Internistische Intensivmedizin, 91154 Roth, Bavaria, Germany

**Keywords:** apelin-36, no-reflow phenomenon, STEMI

## Abstract

**Background:** In patients with ST-segment elevation myocardial infarction (STEMI), apelin is upregulated and exerts cardioprotective effects against ischemia–reperfusion injury (IRI). The present study aimed to investigate serum apelin-36 levels in STEMI patients and their relationship with the no-reflow phenomenon. **Methods:** In this study, 161 patients presenting with STEMI within 12 h of symptom onset and undergoing primary percutaneous coronary intervention (pPCI) were enrolled. Biochemical parameters, including apelin-36, troponin T, creatine kinase (CK), the MB fraction of creatine kinase (CK-MB), total cholesterol, triglycerides, and other routine laboratory parameters, were measured. Two-dimensional echocardiography was performed in all patients. Thereafter, patients were divided into two groups according to their level of aaapelin-36. **Results:** Among the 161 consecutive STEMI patients, 115 (71.42%) had Apelin-36 levels ≤ 0.58 ng/mL (group 1), whereas 46 (28.57%) had Apelin-36 levels > 0.58 ng/mL (group 2). In total, 51 (31.67%) STEMI patients experienced no-reflow phenomenon after PCI: 29 (25.21%) of patients with apelin-36 ≤ 0.58 ng/mL and 22 (47.82%) of those with a value > 0.58 ng/mL (*p* < 0.001). In terms of Gensini score, the mean value in group 1 was 70.29 (±28.76), while in group 2, it was 81.95 (±23.82) (*p* = 0.004). Overall, a positive correlation between apelin-36 and Gensini score was observed in both groups using Kendall’s correlation analysis (group 1: *p* = 0.05; group 2: *p* < 0.0001). Binary logistic regression analysis identified apelin-36 and diabetes mellitus as significant predictors at the 5% level, with *p*-values of 0.045 and 0.036, respectively. Patients with apelin-36 levels ≤ 0.58 ng/mL had troponin T levels of 290.0 (8.5–9510.0), while those with a value > 0.58 ng/mL had troponin T levels of 132.15 (9.4–5190.0) (*p* < 0.012). The receiver operating characteristics (ROC) curve of apelin-36 was used to plot the true positive rate against the false positive rate at different cut-off points, with AUC = 0.77 (95% CI, 0.69–0.84), and the cut-off value for apelin-36 was 0.58 ng/mL, with *p* = 0.001. **Conclusions:** Significant associations were observed between apelin-36 and the no-reflow phenomenon in patients with STEMI. An apelin-36 cut-off value of 0.58 ng/mL, measured at admission, could be used to identify patients who were at increased risk of no-reflow phenomenon/reperfusion injury.

## 1. Introduction

ST-segment elevation myocardial infarction (STEMI) is a life-threatening condition caused by acute coronary occlusion following plaque rupture and subsequent thrombus formation, in which activation of the apelin system may be cardioprotective.

Apelin is encoded by the APLN gene on the X chromosome (Xq-25-q-26.1) [1]. It is synthesized as a 77-amino acid precursor (pre-proapelin) and processed into several biologically active isoforms, including apelin-55, -36, -17, -13 and -12 [2]. Apelin peptides are degraded by plasma kallikrein, neprilysin, and angiotensin-converting enzyme 2 (ACE2) [3,4,5]. Apelin mRNA is widely expressed in blood vessels -endothelial cells, heart-endocardial cells, lungs, kidneys and central nervous system [6,7,8].

Apelin peptides signal via the apelin receptor (APLNR/APJ), a G protein-coupled receptor expressed in cardiovascular and other tissues [9]. In STEMI patients, activation of the apelin/APJ system may influence troponin degradation, as it is known to improve cardiac contractility through increased myofilament calcium sensitivity and the protein kinase C (PKC)–extracellular regulated kinase (ERK1/2) signaling [10,11,12,13].

During STEMI management, reperfusion therapy may induce myocardial ischemia–reperfusion injury (MIRI), including myocardial stunning, no-reflow phenomenon (microvascular damage), reperfusion arrhythmia, and lethal reperfusion injury, mediated by pH paradox, calcium overload, burst of reactive oxygen species (ROS), mitochondrial dysfunction, imbalance of protein phosphorylation, and inflammation [14,15,16,17,18,19].

**Figure 1:**
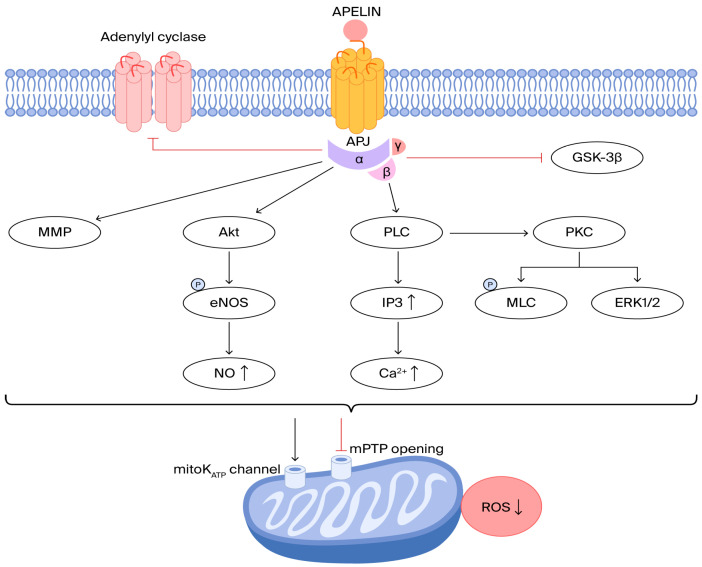
The cardioprotective effects of apelin. APJ: apelin receptor; GSK-3β: glycogen synthase kinase 3β; mPTP: mitochondrial permeability transition pore; PLC: phospholipase C; PKC: protein kinase C; ERK1/2: extracellular regulated kinase; MLC: myosin light-chain; IP3: inositol triphosphate; MMP: matrix metalloproteinase; Akt: protein kinase B; NOS: NO-synthase; ROS: reactive oxygen species.

The cardioprotective effect of apelin during myocardial ischemia–reperfusion is linked to the inhibition of the mitochondrial permeability transition pore, glycogen synthase kinase-3β, and adenylyl cyclase, while it activates several key pathways, such as PI3-kinase, Akt, ERK1/2, NOS, MMP, and the mitoK_ATP_ channel (Figure 1) [20]. Thus, apelin exerts cardioprotective effects in MIRI by reducing ROS, whereas C-reactive protein (CRP) induces ERK1/2 phosphorylation and ROS overproduction [21].

In STEMI patients experiencing the no-reflow phenomenon, apelin levels initially increase and then decline from 24 h onward, potentially contributing to cardioprotection against ischemia–reperfusion injury (IRI) [22,23]. In preclinical models, administration of apelin or elabela (a polypeptide that is also encoded by the APLN gene) at the time of reperfusion protects against IRI, indicating potential for clinical use [24,25]. In addition, Apelin (AP) and Elabela (Ela), components of the apelinergic system, are assumed to modulate endothelial function and contribute to atherosclerosis [26,27].

As far as we know, no study has evaluated the predictive value of apelin-36 level on the no-reflow phenomenon in STEMI patients.

This study aimed to evaluate the relationship between apelin-36 levels and the thrombolysis in myocardial infarction (TIMI) flow grade, as well as to establish a cut-off value for apelin-36 to identify patients at risk for the no-reflow phenomenon.

## 2. Methods

### 2.1. Study Design

Our study was performed as a prospective study, which included 161 consecutive patients who were diagnosed with ST-segment elevation myocardial infarction (STEMI). These patients were enrolled in our center from October 2024 to June 2025. Based on their serum apelin-36 levels (the cut-off value was 0.58 ng/mL), the patients were divided into two groups: group 1 (apelin-36 level ≤ 0.58 ng/mL) and group 2 (apelin-36 level > 0.58 ng/mL). Patients were considered eligible for STEMI if they met all of the following criteria: (1) cardiac biomarker detection (evidence of elevated cardiac troponin (cTn), based on the ESC 0 h/1 h or 0 h/2 h diagnostic algorithms); (2) clinical symptoms (symptoms consistent with myocardial ischemia); (3) electrocardiographic (ECG) changes (new ST-segment elevation at the J-point in at least two contiguous leads, defined as ≥2.5 mm in men < 40 years, ≥2.5 mm in men ≥ 40 years, or ≥1.5 mm in women (regardless of age) in leads V2-V3 and/or ≥1 mm in other leads, as well as in the absence of left ventricle [LV] hypertrophy or left bundle branch block [LBBB]); and (4) patients who underwent primary percutaneous coronary intervention (pPCI) (persistent ST-segment elevation and symptoms of ischemia of ≤12 h) [28]. The exclusion criteria were previous myocardial infarction, thyroid dysfunction, renal insufficiency (estimated glomerular filtration rate (eGFR) < 60 mL/min/1.73 m^2^), inflammatory disease, acute infectious disease, and malignancy.

A comprehensive clinical history was obtained, including assessment of established coronary risk factors (diabetes mellitus, dyslipidemia, arterial hypertension, and smoking), prior pharmacological treatments, and the time elapsed from symptom onset to hospital admission.

Laboratory evaluations comprised measurements of apelin-36, creatine kinase (CK), creatine kinase-MB (CK-MB), cardiac troponin T (cTnT), cholesterol, triglycerides, and standard biochemical parameters. Blood samples were collected approximately 30 min after hospital admission. To measure apelin-36, serum was separated from the blood by centrifugation at 3.000 rpm for 10 min and kept frozen at −80° until analysis. Apelin-36 concentrations were measured using an enzyme-linked immunosorbent assay (ELISA) kit (Phoenix Pharmaceuticals, Inc., Burlingame, CA, USA), following the manufacturer’s protocol (Awareness Technology, ChemWell 2 Automated ELISA, University Clinical Center of Kosova (UCCK), Prishtina, Kosova).

Revascularization was performed in all patients (Coronary angiography, Philips Allura Xper FD20 Cath/Angio System, Ryoal Philips, Best, The Netherlands), accompanied by periprocedural pharmacotherapy in accordance with current guidelines. Post-percutaneous coronary intervention (PCI) management followed standard therapeutic protocols: aspirin (100 mg), clopidogrel (75 mg) or prasugrel (10 mg), β-blockers, lipid-lowering agents, and either angiotensin-converting enzyme (ACE) inhibitors or angiotensin II receptor blockers (ARB_s_), in line with international recommendations [28,29].

Coronary perfusion was assessed angiographically using the Thrombolysis in Myocardial Infarction (TIMI) flow grading system, where TIMI flow grade 0 indicates no contrast flow beyond the occlusion; grade 1 indicates minimal penetration of contrast beyond the lesion without full distal perfusion; grade 2 indicates delayed but complete distal perfusion; and grade 3 indicates normal coronary flow. The no-reflow phenomenon was defined as impaired epicardial coronary flow, indicated by TIMI flow grade 0–2, following removal of coronary obstruction. Final coronary angiographic images obtained immediately after primary PCI were independently assessed by two experienced interventional cardiologists blinded to the apelin-36 levels [30].

Comprehensive transthoracic echocardiography Philips (EPIQ 7C, X5-1 probe, Philips Ultrasound LCC, Bothell, WA, USA) was performed in all patients to quantify left ventricular ejection fraction (LVEF) and assess overall cardiac function.

The study was conducted in accordance with the Declaration of Helsinki and was approved by the Ethics Committees of Medical Faculty-University of Prishtina “Hasan Prishtina” (nr. 9439/2024), and the Kosovo Doctors Chamber (KDC), Prishtina (nr. 228/2024).

### 2.2. Statistical Analysis

The principal aim of this study was to evaluate the relationship between apelin-36 levels and the occurrence of the no-reflow phenomenon in patients presenting with ST-segment elevation myocardial infarction (STEMI). Continuous variables are reported as mean ± SD or median (IQR) for normally and non-normally distributed data. Categorical variables are presented as counts and percentages. Group comparisons were performed using Student’s *t*-test or the Mann–Whitney test for continuous variables and chi-square or Fisher’s exact test for categorical variables. Binary logistic regression was used to identify independent predictors of the outcome. Results are presented as odds ratios (ORs) with 95% confidence intervals (CIs). Model fit was evaluated using the Hosmer–Lemeshow test. Receiver operating characteristic (ROC) curve analysis was performed to assess the discriminative ability of the variables, plotting the sensitivity against the 1-specificity across a range of thresholds. The area under the curve (AUC) was calculated to quantify predictive accuracy for binary outcomes, and the optimal cut-off was determined based on the highest combination of sensitivity and specificity. A two-tailed *p*-value < 0.05 was considered statistically significant. All statistical analyses were performed using SPSS statistical software, version 26.

## 3. Results

**Figure 2:**
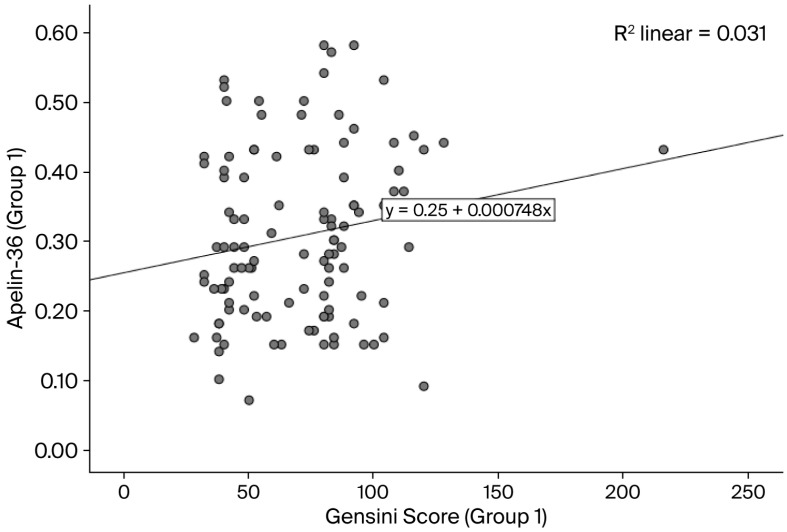
Correlation between apelin-36 levels and Gensini score in group 1 (Kendall’s correlation coefficient r = 0.12; *p* = 0.05).

**Figure 3:**
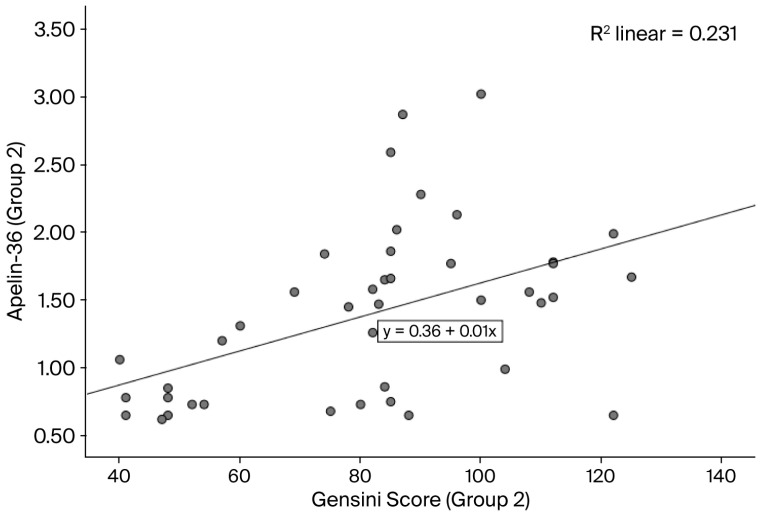
Correlation between apelin-36 levels and Gensini score in group 2 (Kendall’s correlation coefficient r = 0.39; *p* < 0.0001).

The baseline characteristics of the study population are summarized in Table 1. In this study, we included 161 consecutive STEMI patients: 115 (71.42%) with apelin-36 ≤ 0.58 ng/mL (group 1) and 46 (28.57%) with a value > 0.58 ng/mL (group 2). In group 1, the mean age was 62.61 ± 10.95 years, and 96 (83.47%) of the patients were male, while in group 2, the mean age was 59.37 (±11.30) years, and 36 (78.26%) of the patients were male. The comparison of diabetes mellitus, creatinine, and troponin T between groups with different levels of apelin-36 (≤0.58 ng/mL and >0.58 ng/mL) was statistically significant (the *p*-value was 0.006 for diabetes mellitus, 0.014 for creatinine, and 0.012 for troponin T). In terms of the Gensini score, the mean value in group 1 was 70.29 (±28.76), while in group 2, it was 81.95 (±23.82) (*p* = 0.004). A positive correlation between apelin-36 and Gensini score was observed in both groups using Kendall’s correlation analysis (group 1: Figure 2, *p* = 0.05; group 2: Figure 3, *p* < 0.0001). In total, 51 (31.67%) of the STEMI patients experienced no-reflow phenomenon after PCI: 29 (25.21%) of patients with apelin-36 ≤ 0.58 ng/mL and 22 (47.82%) of those with a value > 0.58 ng/mL (*p* = 0.001).

**Table 1:** Baseline characteristics according to apelin-36 level.

Characteristics	Apelin-36 ≤ 0.58 ng/mL (n = 115)	Apelin-36 > 0.58 ng/mL (n = 46)	*p*-Value
Age (year)	62.61 (±10.95)	59.37 (±11.30)	0.22
Gender (male) (n/%)	96 (83.47)	36 (78.26)	0.80
BMI (kg/m^2^)	27.28 (±5.32)	26.24 (±5.64)	0.96
Medical history			
Hypertension (n/%)	66 (57.39)	24 (52.17)	0.61
Diabetes mellitus (n/%)	35 (30.43)	10 (21.73)	0.006
Smoking (n/%)	69 (60.0)	27 (58.69)	0.54
Laboratory values			
Hemoglobin (mg/dL)	138.55 (±23.68)	141.0 (±15.49)	0.99
Cholesterol (mmol/L)	5.17 (1.16)	5.02 (1.49)	0.33
Triglyceride (mmol/L)	1.75 (±1.12)	1.52 (±0.75)	0.43
Glucose (mmol/L)	10.27 (±5.77)	8.48 (±4.21)	0.02
Creatinine (umol/L)	105.73 (31.27)	92.95 (26.97)	0.014
Creatine kinase-MB (U/L)	124.0 (15.0–466.0)	90 (16.0–520.0)	0.17
Creatine kinase (U/L)	431.0 (210–7224.0)	752.50 (350.0–5057.0)	0.61
Troponin T (pg/mL)	290.0 (8.5–9510.0)	132.15 (9.4–5190.0)	0.012
Ejection fraction (%)	49.43 (±6.55)	50.30 (±7.14)	0.44
Gensini score (Mean ± s.d)	70.29 (±28.76)	81.95 (±23.82)	0.004
Final TIMI grade flow ≤ 2 (n/%)	29 (25.21)	22 (47.82)	0.001

BMI: body mass index; TIMI: thrombolysis in myocardial infarction.

Based on binary logistic regression (Table 2), we analyzed the relationship between one dichotomous dependent variable (TIMI flow grade) and other variables (BMI, diabetes mellitus, smoking, apelin-36, creatine kinase-MB, creatine kinase, and troponin T). Apelin-36 and diabetes mellitus are significant predictors at the 5% level, with *p*-values of 0.045 and 0.036. Predictors such as BMI, smoking, creatine kinase, creatine kinase-MB, and troponin T, with *p*-values above 0.05, are not significant predictors of the TIMI flow grade at the 5% level.

**Table 2:** Binary logistic regression analysis of no-reflow phenomenon.

Parameter	OR	95% CI	*p*-Value
BMI (kg/m^2^)	0.89	0.69–1.16	0.41
Diabetes mellitus (n/%)	0.07	0.007–0.85	0.036
Smoking (n/%)	4.96	0.76–32.10	0.09
Apelin-36 (ng/mL)	0.038	0.002–0.92	0.045
Creatine kinase-MB (U/L)	1.003	0.99–1.01	0.57
Creatine kinase (U/L)	0.99	0.99–1.00	0.15
Troponin T (pg/mL)	1.00	1.00–1.001	0.16

OR: odds ratio; BMI: body mass index.

A receiver operating characteristic (ROC) curve of apelin-36 was used to plot the true positive rate against the false positive rate across varying cut-off points; the area under the curve (AUC) was 0.77 (95% CI, 0.69–0.84), while the cut-off value for apelin-36 was 0.58 ng/mL, with *p* < 0.0001.

Table 3 presents the AUC values of biochemical parameters other than apelin-36.

**Table 3:** Area under the curve values for biochemical analysis.

Parameter	AUC (95% CI)	*p*-Value
Apelin-36	0.77 (0.69–0.84)	<0.0001
Creatine kinase	0.57 (0.34–0.80)	0.49
Creatine kinase-MB	0.59 (0.37–0.80)	0.40
Troponin T	0.57 (0.36–0.78)	0.49
Na^+^	0.41 (0.20–0.62)	0.41
K^+^	0.55 (0.33–0.77)	0.61
Ca^++^	0.51 (0.31–0.72)	0.88
Cholesterol	0.70 (0.51–0.58)	0.07
Triglyceride	0.42 (0.20–0.64)	0.47
Hemoglobin	0.59 (0.37–0.81)	0.37
Glucose	0.67 (0.48–0.86)	0.10
BUN	0.60 (0.40–0.80)	0.33
Creatinine	0.59 (0.36–0.81)	0.40

AUC: area under the curve; BUN: Blood Urea Nitrogen.

## 4. Discussion

This study investigated the predictive value of apelin-36 in relation to the occurrence of the no-reflow phenomenon in STEMI patients.

The role of apelinergic peptides in the pathogenesis of myocardial ischemia–reperfusion injury (MIRI) is yet to be elucidated. However, many studies have reported that apelin activity may exert a beneficial effect in STEMI patients due to its cardioprotective potential.

The rupture of an atherosclerotic plaque represents the first step in the sequence of events leading to thrombotic coronary occlusion and subsequent myocardial infarction. The oxygen supply to cells is reduced due to impaired blood flow, ultimately resulting in cardiac hypoxia or ischemia [31,32].

Intervention, such as primary PCI, to restore coronary blood flow, is essential for myocardial salvage. Nevertheless, reperfusion can aggravate myocardial damage. This phenomenon, known as ischemia–reperfusion (I/R) injury, encompasses a series of events—including pH paradox, calcium overload, burst of reactive oxygen species (ROS), mitochondrial dysfunction, imbalance of protein phosphorylation, and inflammation—that ultimately lead to myocardial damage, such as myocardial stunning and no-reflow phenomenon (microvascular damage). Following reperfusion injury, excessive ROS production contributes to endothelial dysfunction, deoxyribonucleic acid (DNA) damage, and localized inflammation, ultimately resulting in cellular death. Garciarena et al. demonstrated that reperfusion injury results from ROS-triggered activation of the mitogen-activated protein kinase (MAPK) pathway, which mediates Na^+^/H^+^ exchanger-1 (NHE-1) phosphorylation and reactivation during reperfusion [33]. Also, high circulating levels of CRP are implicated in the progression of reperfusion injury. The pathogenic role of CRP in MIRI is mediated through ERK1/2 signaling, leading to enhanced generation of ROS and intracellular Ca^2+^ accumulation. ERK1/2, a member of the mitogen-activated protein kinase (MAPK) family of serine/threonine kinases, displays dysregulated expression in the setting of MIRI [21]. Approximately 30% of patients are estimated to develop I/R injury, which significantly worsens their prognosis [34,35].

**Figure 4:**
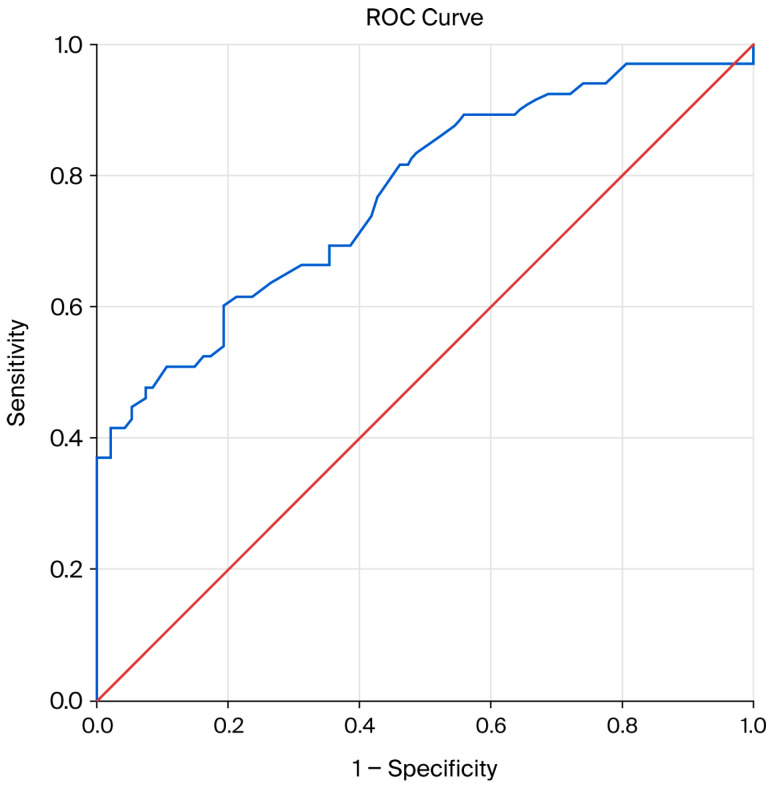
ROC curve analysis of apelin-36 for the prediction of no-reflow phenomenon in STEMI patients. AUC = 0.77 (95% CI, 0.69–0.84); *p* < 0.0001.

Regarding signaling mechanisms, as an APJ agonist, apelin protects against I/R injury, mainly by engaging the Akt/No and ERK1/2 pathways and delaying mPTP opening (downregulation of reactive oxygen species (ROS)) (Figure 1). In our study, the use of ROC curve analysis of apelin-36 for the prediction of no-reflow phenomenon (unsuccessful reperfusion, TIMI flow grade ≤ 2) was statistically significant, with AUC = 0.77 (95% CI, 0.69–0.84) and *p* < 0.0001, confirming its influence on myocardial ischemia–reperfusion injury (MIRI) (Figure 4) [36,37,,38,39,40,41]. Thus, in our study, patients with apelin-36 levels ≤ 0.58 ng/mL showed a significantly higher incidence of the no-reflow phenomenon compared with those with apelin-36 levels > 0.58 ng/mL, with *p* = 0.0001 (Table 1). Based on binary logistic regression analysis, apelin-36 is a significant predictor of the TIMI flow grade at the 5% level, with OR = 0.038, CI of 0.002–0.92, and *p* = 0.045 (Table 2). This study supports the hypothesis that lower apelin-36 concentrations may be associated with MIRI, consistent with preclinical observations. Simpkin et al. were the first to advance the concept that apelin may play a protective role in mitigating reperfusion-induced injury [22]. Also, An et al. provided compelling evidence that apelin confers cardioprotection in diabetic myocardium during ischemia–reperfusion by suppression of apoptotic processes and oxidative stress via phosphoinositide 3-kinase (PI3K) and p-38-MAPK-dependent signaling mechanisms [39]. Wang et al. demonstrated that the absence of apelin increases susceptibility to infarction and worsens cardiac function in both ex vivo and in vivo I/R models [25]. Apelin has recently been proposed as a potential therapeutic target in myocardial I/R injury, given its reported involvement in mechanisms implicated in the condition [42]. The study by Pisarenko et al. was the first to demonstrate that structural analogs of apelin attenuate excessive mitochondrial ROS generation and maintain myocardial metabolic homeostasis in cultured cardiomyocytes as well as in isolated rat heart models subjected to IRI [43].

Apelin improves myocardial contractility by binding to the APJ receptor and activating downstream signaling pathways, including PI3K/Akt and ERK1/2, which augment calcium availability and sensitivity [10]. Farkasfalvi et al. showed that the inotropic effect of apelin may be due to increased myofilament Ca^2+^ sensitivity, associated with Na^+^/H^+^ exchanger (NHE) activation and intracellular alkalinization [44]. Myocardial infarction is characterized by decreased phosphorylation of cardiac troponin (cTn), reduced Ca^2+^ sensitivity, and diminished ATPase activity. Elevated cytosolic Ca^2+^ subsequently triggers protease I (calpain I) activation, leading to proteolytic degradation of troponins. Concurrently, apelin and APJ expression are upregulated, a process that is believed to exert cardioprotective effects by attenuating ischemic myocardial damage (degradation of troponin) [12,45,46,47,48]. In our study, patients with apelin-36 levels ≤ 0.58 ng/mL showed significantly higher levels of troponin T than those with apelin-36 levels > 0.58 ng/mL (*p* = 0.012), confirming the role of apelin in reducing troponin degradation (Table 1). Babapour et al. also demonstrated the role of apelin in relation to troponin levels. They reported a significant negative correlation between apelin and troponin-T (r = −0.288, *p* = 0.006) [49]. Tang et al. developed a cardiac patch embedded with apelin-formulated microparticles to treat myocardial infarction in mice [11].

Apelin and APJ receptor expressions are increased in human coronary atherosclerotic plaques, co-localizing with inflammatory macrophages and vascular smooth muscle cells, which indicates their involvement in plaque progression and vascular remodeling [50]. Through ERK activation, apelin/APJ signaling elevates endothelial intercellular adhesion molecule-1 (ICAM-1) and vascular cell adhesion molecule-1 (VCAM-1) levels and stimulates monocyte chemoattractant protein-1 (MCP-1) release via the NF-κB/JNK pathway, thereby facilitating monocyte recruitment and adhesion—key events in early atherogenesis [51,52]. Li et al. demonstrated that apelin attenuates diabetes mellitus-induced microvascular dysfunction by improving endothelial function through APJ receptor-dependent regulation of NF-κB signaling pathways [53].

In patients with stable coronary artery disease (CAD), plasma apelin levels are reduced and inversely associated with the severity of coronary lesions, showing a negative correlation with the Gensini score [54,55]. Li et al. reported reduced apelin levels in patients with stable angina, which were inversely associated with the degree of coronary stenosis (Gensini score) (r = −0.399, *p* < 0.05) [55]. Also, Namazi et al. observed that serum apelin levels were inversely associated with the presence and severity of coronary artery disease [50]. Apelin upregulation during acute coronary syndrome (ACS) shows a positive correlation with the Gensini score, suggesting a compensatory cardioprotective role [56]. In our study, the Gensini score was significantly lower in group 1 (apelin-36 ≤ 0.58 ng/mL) than in group 2 (apelin-36 > 0.58 ng/mL) (*p* = 0.004) (Table 1). Overall, apelin-36 levels were positively correlated with the Gensini score. Specifically, Kendall’s correlation analysis demonstrated a weak positive correlation in group 1 (r = 0.12; *p* = 0.05) and a moderate positive correlation in group 2 (r = 0.39; *p* < 0.0001) (Figure 2 and Figure 3). Sheng-Li et al. reported that Elabela, as part of the apelin/Elabela-APJ axis, was positively correlated with the severity of coronary stenosis in patients with acute coronary syndrome [57].

This result suggests that apelin plays a cardioprotective role and that its upregulation following ACS-STEMI may have therapeutic potential, particularly in no-reflow phenomenon/myocardial ischemia–reperfusion injury.

We acknowledge certain limitations of our study. Assessing apelin levels in control groups of patients without ST-segment elevation myocardial infarction (NSTEMI) and patients with stable angina would strengthen a comparative analysis. In addition, the absence of glycated hemoglobin (HbA1C) and uric acid represents a further limitation. Other limitations include the absence of correlation of apelin-36 with the severity of coronary lesions and the location of myocardial infarction, as well as the single-center study design and the moderate sample size.

## 5. Conclusions

Serum apelin-36 levels are significantly associated with the no-reflow phenomenon in patients with STEMI undergoing primary percutaneous coronary intervention. Apelin-36 was identified as an independent predictor of impaired coronary flow and demonstrated good discriminative ability for no-reflow, with a cut-off value of 0.58 ng/mL and superior predictive performance compared with traditional biomarkers. Furthermore, apelin-36 levels were positively correlated with the Gensini score. These findings suggest that apelin-36 may serve as a useful early biomarker for identifying STEMI patients at increased risk of microvascular dysfunction, as well as showing potential therapeutic relevance. Further large-scale studies are needed to validate its prognostic value and to explore the therapeutic potential of targeting the apelinergic system in ischemia–reperfusion injury.

## Data Availability

The data presented in this study are available from the corresponding author upon reasonable request due to privacy and ethical considerations.
